# STAT3 Contributes to Radioresistance in Cancer

**DOI:** 10.3389/fonc.2020.01120

**Published:** 2020-07-07

**Authors:** Xuehai Wang, Xin Zhang, Chen Qiu, Ning Yang

**Affiliations:** ^1^Department of Otolaryngology, Weihai Municipal Hospital, Shandong University, Weihai, China; ^2^Department of Neurosurgery, Qilu Hospital of Shandong University and Institute of Brain and Brain-Inspired Science, Shandong University, Jinan, China; ^3^Shandong Key Laboratory of Brain Function Remodeling, Jinan, China; ^4^Department of Radiation Oncology, Qilu Hospital of Shandong University, Jinan, China

**Keywords:** STAT3, apoptosis, aggressive behaviors, cancer cell stemness, reactive oxygen species

## Abstract

Radiotherapy has been used in the clinic for more than one century and it is recognized as one of the main methods in the treatment of malignant tumors. Signal Transducers and Activators of Transcription 3 (STAT3) is reported to be upregulated in many tumor types, and it is believed to be involved in the tumorigenesis, development and malignant behaviors of tumors. Previous studies also found that STAT3 contributes to chemo-resistance of various tumor types. Recently, many studies reported that STAT3 is involved in the response of tumor cells to radiotherapy. But until now, the role of the STAT3 in radioresistance has not been systematically demonstrated. In this study, we will review the radioresistance induced by STAT3 and relative solutions will be discussed.

## Introduction

With the development of technology and our understanding of tumors, there are multiple methods for the treatment of tumors, such as surgical resection, chemotherapy, radiotherapy and immunotherapy. Among all these approaches to treatment, radiotherapy is one of the most cost-effective methods for the treatment of various cancers. Since its introduction into the clinic, radiotherapy has existed for more than one century, which shows that its efficiency is well-approved (1). But for some tumor types, radiotherapy doesn't work so well. Until now, we have recognized that after irradiation treatment, tumor cells develop complicated mechanisms, like DNA repair, cell cycle arrest, and autophagy, to protect themselves so as to survive (2–4). As a result, most tumor cells will develop radioresistance, which leads to the failure of radiotherapy. Although many methods have been used to overcome radioresistance, their efficiency is not as what we have expected (5, 6). Thus, more research is needed to help us have a better understanding of radioresistance.

Signal Transducers and Activators of Transcription 3 (STAT3), one of the most important intracellular transcription factors, is reported to be constitutively activated in most tumor types (7, 8). Many cytokines, hormones and growth factors are involved in the activation of STAT3, among which canonical Janus kinase (JAK) is the most studied (9). STAT3 plays an important role in cell proliferation, and is also involved in anti-apoptosis process (9). Besides, STAT3 also promotes angiogenesis, invasiveness and immunosuppression in cancer (10–12). All these functions are related to STAT3's role in controlling gene transcription. Previously, STAT3 was recognized as a direct transcription factor. For example, STAT3 regulates pro-survival gene expression to increase apoptotic resistance in cancer. But more and more recent studies discover that STAT3 also regulates gene expression through DNA methylation and chromatin modulation (9).

Previously, STAT3 is reported to be involved in chemo-resistance and this is well-reviewed by Tan et al. (13). Recently, more and more studies showed that STAT3 contributed to radioresistance. Huang et al. showed that sorafenib and its derivative could increase the anti-tumor effect of radiotherapy by inhibiting STAT3 (14, 15). Lau et al. found that blocking STAT3 could inhibit radiation-induced malignant progression, such as increased migration and invasion (16). These studies have shown that STAT3 is not only involved in tumorigenesis and tumor development, but also leads to chemo- and radio-resistance. Here, we mainly review the role of STAT3 in mediating radioresistance from points of apoptosis, aggressive behaviors, DNA damage repair, cancer stem cells. And novel modalities to reverse the failure of radiotherapy will be discussed.

## Inhibiting STAT3 Increased Radiation-Induced Apoptosis

Apoptosis is essential for the maintenance of normal physiological functions in normal cells. But anti-apoptosis process could be induced in tumor cells after chemo- or radio-therapy, so as to help them survive (17).

The B cell lymphoma-2 (BCL-2) family of proteins are important among all the factors that are involved in regulating programmed cell death or apoptosis (18). BCL-2 family of proteins are mainly mitochondria localized and involved in the release of cytochrome C, an essential mediator of apoptosis (19, 20). They possess BH 1–4 domains and have the function of maintaining mitochondrial integrity. STAT3 is involved in tumorigenesis by activating anti-apoptotic proteins, like surviving (17, 21). Conversely, inhibiting STAT3 increases apoptosis (22, 23). Besides, a study found that heat shock protein 70 (Hsp70) has anti-apoptosis effect by preventing JNK-induced phosphorylation and inhibiting BCL-2 and BCL-XL, so as to maintain the stability of mitochondria (24). STAT3 can affect the Hsp70 promoter and increases its expression in cancer cells, mediating the upregulation of antiapoptotic proteins (25). As a result, we assume that inhibiting STAT3 could decrease the expression of antiapoptotic proteins, leading to apoptosis.

Until now, there are a large number of studies showing that inhibiting STAT3 increases radiosensitivity in numerous tumor types (14, 15, 26–31), which are summarized in Table 1.

**Table 1:** A summary of pre-clinical studies in which STAT3-targeted compounds are used to enhance the radiosensitivity of malignant tumors by inducing apoptosis.

**Compounds/Genes**	**Cancer type**	**Mechanisms**	**References**
Sorafenib	Hepatocellular carcinoma	Inhibiting STAT3	(14)
SC-59	Hepatocellular carcinoma	Inhibiting SHP-1/STAT3 pathway	(15)
Dovitinib	Hepatocellular Carcinoma	Inhibiting SHP-1/STAT3 pathway	(26)
GRIM-19	Gastric cancer cells	Suppressing accumulation of STAT3	(27)
Stattic	Head and neck squamous cell carcinoma	Inhibiting STAT3	(28)
Casticin	Human renal clear cell carcinoma, neck squamous cell carcinoma	Inhibiting IL-6/STAT3 pathway	(29)
Zoledronic acid	Human pancreatic cancer cells	Inhibiting STAT3/NF-kappa B pathway	(30)
YM155	Glioma	Inhibiting STAT3/survivin pathway	(32)

*GRIM-19, genes associated retinoid–IFN induced mortality-19. SHP-1, Src-homology phosphatase type-1*.

Studies showed that STAT3 is one of the most important regulators of survivin (33, 34). Survivin, a member of the inhibitor of apoptosis protein (IAP) family and one of the most important anti-apoptotic proteins, is found highly expressed in various cancer types, making it a potential anticancer target (35, 36).

Several studies found that inhibiting survivin could enhance radiosensitivity in various tumor types. Grdina et al. found that the expression of survivin upregulated after irradiation treatment and increased survivin promoted cell survival, while knocking down survivin with siRNA abrogated this adaptive response (37). Iwasa et al. showed that YM155, an inhibitor of survivin, enhanced radiosensitivity in non-small cell lung cancer cell lines (38). Qin et al. showed that YM155 increases radiosensitivity in esophageal squamous cell carcinoma by the inhibiting cell cycle checkpoint and homologous recombination repair (32).

But these studies didn't demonstrate whether the radiosensitive effect of YM155 is STAT3-dependent. Our recent study found that YM155 decreased the activity of STAT3 and increased the radiosensitivity of glioblastoma, one of the most radioresistant tumor types. This might provide some clues for the role of STAT3/survivin pathway in the radioresistance of various neoplasms (31). Further studies are warranted to clarify the direct interaction between STAT3 and survivin after irradiation treatment.

## Inhibiting STAT3 Decreased Radiation-Induced Aggressive Behaviors

Although irradiation kills cancers, we have to recognize that it can also induce carcinogenesis. The carcinogenic potential of ionizing radiations could be traced back to 1902 and more radiation-induced (RI) neoplasia have been observed (39, 40). Over the last 45 years, 296 cases of radiation-induced brain tumors have been reported.

Except for RT tumors, more and more studies discovered that radiation might enhance malignant progression, like aggressive migration and invasion in cancer cells (16, 41–45). This will induce more extensive spread of tumor cells, which is a contributing factor to tumor relapse and recurrence (46, 47).

Among all the mechanisms that explaining radiation-induced migration and invasion, epithelial–mesenchymal transition (EMT) accounting for the most. In the process of EMT, epithelial characteristics will be downregulated and mesenchymal characteristics will be gained in epithelial cells. This phenomena were reported by Elizabeth Hay in the early 1980s (48). Cancer cells underwent EMT will acquire elevated capabilities to invade and disseminate to distant sites, which increases its malignance.

Previous studies showed that among all signaling pathways that are involved in EMT, STAT3 is one of the most important (49–51). Many studies found that irradiation actually promoted EMT process (43, 52, 53). Lau et al. found that blocking STAT3 decreased radiation-induced malignant behaviors in glioma (16). Our previous study showed that YM155 not only decreased DNA damage repair, but also decreased radiation-induced invasion and reversed EMT by inhibiting STAT3, which was a promising radiosensitizer in the treatment of cancer (31). But whether it has the same effect in other tumor types is still unknown.

Except for EMT process, there are other mechanisms to explain for the increased invasive ability induced by irradiation (42, 54–61), which are summarized in Table 2. But whether they have relationships with STAT3 is still unknown.

**Table 2:** A summary of genes involved in irradiation-induced invasion of various cancers.

**Genes**	**Cancer type**	**Mechanisms**	**References**
MMP2	Glioma; Esophageal squamous cell carcinoma	Degrading extracellular matrix components	(42, 54, 55)
SDF-1	Murine astrocytoma tumor	Through macrophage mobilization and tumor revascularization	(56)
MRCK	Glioma; Squamous cell carcinoma; Skin cancer	By targeting MLC and MYPT1; By disturbing a network of communicating glioma cell protrusions	(57–60)
MMP9	Glioma	Degrading extracellular matrix components	(61)

*MLC, myosin light chain proteins. MRCK, myotonic dystrophy kinase-related CDC42-binding kinase. MMP: matrix metalloproteinase. MYPTI, myosin phosphatase targeting subunit 1. SDF-1: stromal cell-derived factor-1*.

Yu et al. showed that irradiated breast cancer cells could promote the invasion of non-irradiated tumor cells and angiogenesis through IL-6/STAT3 signaling (62). This might explain that STAT3 plays a role in radiation-induced bystander effect (RIBE) (63). RIBE happens when irradiated cells affect non-irradiated cells through gap junctional intercellular communication (GJIC) or the release of soluble factors (64). Results induced by RIBE are still unclear. Some studies found decreased survival in unirradiated cells, whereas others observed that RIBE helped irradiated cells survive (65).

Duan et al. showed that irradiation of normal brain before tumor cell implantation contributed to aggressive tumor growth, which suggested a brain tumor microenvironment-induced, tumor-extrinsic effect (66). Tumor microenvironment (TM) is mainly composed of epithelial cells, stromal cells, extracellular matrix (ECM) components or immune cells (67). The concept of TM can be traced back to 1889, when Paget put forward the concept, “seed and soil” (68). The role of TM in tumorigenesis and development is attracting more and more attention. Studies also found that TM contributed to chemo- or radio-resistance (67, 69, 70).

Recent studies also verified the important role of STAT3 in tumor microenvironment. A study by Chang et al. concluded that IL-6/JAK/STAT3 pathways played a key role in regulating the tumor microenvironment that promoted growth, invasion, and metastasis (71). Deng et al. reported that sphingosine-1-phosphate receptor type 1 (S1PR1)-STAT3 signaling was activated in pre-metastatic sites, contributing to the formation of pre-metastatic niche (72). Bohrer et al. found that upregulation of the fibroblast growth factor receptor (FGFR)-STAT3 signaling in breast cancer cells led to a hyaluronan-rich microenvironment, which helped tumor progression (73).

STAT3 also established an immunosuppressive microenvironment to promote the growth and metastasis breast cancer (74). All these studies showed that STAT3 played an important role in tumor microenvironment-induced aggressive behaviors, which made STAT3 a promising target in the radiorensitization of cancers. A study by Gao et al. showed that myeloid cell-specific inhibition of Toll-like receptor 9 (TLR9)/STAT3 signaling enhanced the antitumor effect of irradiation (75). More studies are warranted in the future to verify whether targeting STAT3 is efficient in inhibiting irradiation-induced malignant behaviors of cancers.

## STAT3 is Involved in DNA Damage Repair

Ionizing radiation damages DNA mainly in two ways, by direct and indirect action. Direct action occurs by ionization in the DNA molecule itself. On the other hand, ionization of water produces reactive oxygen species (ROS) and reactive nitrogen species (RNS), and these free radical species (in particular the OH radical) damage DNA by indirect action (31). The efficacy of radiotherapy is mainly determined by DNA damage. However, radioresistance can be induced after repeat radiotherapy treatment. Among all the cellular processes that are involved in the development of radioresistance, DNA damage repair is one of the most important factors (76).

When DNA damage occurs, a series of sequential reactions are induced to maintain the consistency and integrity of genetic material. These reactions are called DNA damage repair (77). Among all the pathways that are involved in DNA damage repair, ataxia-telangiectasia mutated (ATM)- check-point kinase 2 (Chk2) and ATM and Rad3-related (ATR)- check-point kinase 1 (Chk1) signaling pathways are the best studied. ATM and ATR are recognized as the central components of the DNA damage response (78, 79). When DNA double strand breaks (DSBs) occur, ATM is upregulated and activates the phosphorylation of important proteins like check-point kinase 2 (Chk2), p53, and BRCA1 (80, 81). ATR initiates the late phosphorylation of p53 and check-point kinase 1 (Chk1) (82, 83). Details can be seen in the review by Zhang et al. (84).

There are various types of DNA damage, like base damage, deletion, insertion, exon skipping, single strand breaks and double strand breaks (DSBs). Among them, DSBs are the most lethal. There are mainly two mechanism in repairing DSBs, non-homologous end joining (NHEJ) and homologous recombination (HR). In the process of NHEJ, ligation occurs regardless of whether the ends come from the same chromosome. As a result, mistakes might occur. On the other hand, HR uses the information that is usually from the sister chromatid to repair damaged DNA. So, HR has a higher accuracy in repairing DNA (76).

Recently, more and more studies showed that STAT3 was involved in DNA damage repair. STAT3 was reported to be involved in the regulation of breast cancer susceptibility gene 1 (BRCA1), an important factor in DNA damage repair, especially HR (85, 86). Barry et al. showed that knocking down STAT3 impaired the efficiency of damage repair by downregulating the ATM-Chk2 and ATR-Chk1 pathways (87). Essential meiotic endonuclease 1 homolog 1 (Eme1), a key endonuclease involved in DNA repair, was reported to be the downstream of STAT3 (88–90).Chen et al. showed that silencing Jumonji domain-containing protein 2B (JMJD2B) activated DNA damage by the suppression of STAT3 signaling (91). All these studies suggested that STAT3 was part of DNA damage repair mechanisms. But it is still insufficient to conclude that STAT3 is directly participating in DNA damage repair. What's more, these studies are not enough to show that it is ubiquitous for STAT3 to be involved in DNA damage repair. Studies like knocking down STAT3 to test the efficiency of HR or NHEJ are needed in the future.

## STAT3 Contributes to ROS Depletion in Cancer Stem Cells

Cancer stem cells (CSCs) are a group of cells with characteristics of self-renewing, multipotent, and tumor-initiating. Recently more and more studies showed that most malignant cancer cells were derived from CSCs (92, 93). CSCs have high expression of anti-apoptotic proteins and ATP-binding cassette (ABC) pump, and all these contribute to its chemo- or radio-resistant feature (94–96). Many studies showed that STAT3 was one of the most important factors in maintaining the phenotype of CSCs (97–99). Besides, STAT3 also suppressed differentiation-related genes (100). In the aspect of radiotherapy, recent studies found that STAT3 and CCS were closely connected in contributing to radioresistance. Lee et al. found that STAT3 was involved in enhancing cancer stemness and radioresistant properties (101). Shi et al. showed that ibrutinib, a Bruton's tyrosine kinase (BTK) inhibitor, could impair radioresistance by inactivating STAT3 in glioma stem cells (102). A study by Park et al. showed that JAK2/STAT3/ cyclin D2 (CCND2) pathway promoted colorectal cancer stem cell persistence and radioresistance (103). Gao et al. found that lemonin, a triterpenoid compound, enhanced radiosensitivity by attenuating Stat3-induced cell stemness (104). Many studies have been done to find out the underlying mechanisms of CSCs in radioresistance. A review by Skvortsova et al. showed that the ability of DNA damage repair was enhanced in CSCs, so as to help them defend against ROS (105). Arnold found that radiation expanded cancer stem cell populations and can induce stemness in nonstem cells in a STAT3-dependent manner (106). Since ionizing radiation causes cell death by DNA damage, here we mainly focus on how CSCs respond to DNA damage. Until now, little is known about the DNA repair mechanisms in CSCs. But more and more attention has been paid to the role of ROS in CSC survival and radiation resistance (107).

High ROS levels affect many aspects of tumor biology and one of the most important roles is that it induces DNA damage and genomic instability (108). Normally, single strand breaks (SSBs) are the main DNA damage type after ROS treatment and can be repaired through nucleotide or base excision repair (NER/BER) (109). But the accumulation of SSBs can lead to stalling of the replication fork or error in replication, which ultimately induces more lethal DNA damage type, DSBs. A study showed that CSCs possess lower concentrations of ROS than do non-stem cancer cells (96). Studies also found that inhibiting ROS scavenging machinery could enhance radiosensitivity in CSCs (110, 111). Lu et al. found that niclosamide, an inhibitor of STAT3, increased the radiosensitivity in triple negative breast cancer (TNBC) cells via triggering the production of ROS (112). Their study showed that inhibiting STAT3 and increasing ROS led to radiosensitivity. But they didn't illustrate enough evidence to prove the relationship between STAT3 and ROS in DNA damage repair.

Intracellular ROS is mainly produced by the mitochondria, and another source is NADPH oxidases (NOXs) (113). Initially, mitochondria ROS production is unwanted by cells. Recently, more and more studies found that STAT3 was actively involved in regulating the activity of mitochondria. Lapp et al. found that activating STAT3 decreased mitochondrial ROS production by upregulating the expression of uncoupling protein 2 (UCP2) (114). Meier et al. showed that phosphorylation of Ser^727^ in STAT3 recruited mitochondrially localized STAT3 (115). But phospho-Stat3Y^705^ is not responsible for the STAT3 mitochondrial translocation (115–117). What's more, a recent study by Cheng et al. found that selectively inhibiting mitochondrial STAT3 could provide a promising target for chemotherapy (118). As a result, we assume that mitochondrial STAT3 may be a potential target in enhancing the radioresensitizing effect of cancer cells. More studies are warranted in the future.

## Conclusion

Persistent activation of STAT3 in various tumor types makes STAT3 a specific and promising target in anticancer treatment. Inhibiting STAT3 by STAT3 dominant negative molecules, decoy oligonucleotides, and peptidomimetics is proven efficient in numerous preclinical studies.

STAT3 is well-known for its roles in tumor initiation and development. Besides, STAT3 also leads to chemo- or radio-resistance. In this review, we mainly focused on the role of STAT3 in response to radiotherapy, and the underlying mechanisms including but not limited to apoptosis, aggressive behaviors, DNA damage repair, cancer stem cells were discussed (Supplementary Figure 1). Except for the mechanisms we have discussed above, there are still other explanations for STAT3's role in radioresistance. Hypoxia is also a well-known factor that contributes to radioresistance in many tumor types. Hypoxia induces the production of ROS by mitochondrial electron transport chain. It also activates Hypoxia inducible factors (HIFs), important factors which will induce malignant behaviors and proliferation of tumor cells under hypoxia. Mitochondrial ROS stabilizes HIF1 and HIF2 by inhibiting prolyl hydroxylases (PHDs) (119, 120). Studies showed that NSC74859 and stattic, two inhibitors of STAT3, enhanced radiosensitivity by inhibiting hypoxia- and radiation-induced STAT3 activation in esophageal cancer (121, 122).

On the other hand, we have to realize that STAT3 has many other functions except for disease formation and progression, like cardioprotection, liver protection, and obesity (123–126). As a result, targeting STAT3 may have many side effects. For example, strong inhibitors of STAT3 could cause fatigue, diarrhea, infection, and periphery nervous system toxicities (127).

Although the inhibitors of STAT3 have been studied and proven to be efficient in preclinic for 20 years, they showed poor anti-tumor effect in clinical trials (17). Recently, drug repurposing, a method based on the theory that established drugs may have many other mechanisms except for their well-known indications, has gained increased attention (128). Drug repurposing has advantages such as highly approved safety, avoiding laborious and expensive drug development processes. As a result, testing FDA-approved drugs may help us find potential inhibitors of STAT3 and promote its quick translation into clinic to treat human cancers. In conclusion, the role of STAT3 in the radio-response of cancer has been paid more and more attention to. STAT3 is becoming a promising target in the radiosensitization of cancer.

## Author Contributions

XW, XZ, and NY contributed to conception and manuscript writing. XW and CQ searched the literature. NY supervised the whole writing work. All authors have read and approved the final manuscript.

## Conflict of Interest

The authors declare that the research was conducted in the absence of any commercial or financial relationships that could be construed as a potential conflict of interest.

## Supplementary Material

Supplementary Figure 1 Radioresistance caused by signaling pathways related to STAT3. Radiation induced anti-apoptosis is mediated by STAT3-HSP70-BCL2 family members pathways. After radiation treatment, STAT3 promoted aggressive behaviors in tumor cells through epithelial–mesenchymal transition (EMT), radiation-induced bystander effect (RIBE) and tumor microenvironment (TM). STAT3 contributes to DNA damage repair through ATM-Chk2 and ATR-Chk1 pathways. Cancer stemness and reactive oxygen species (ROS) depletion are also involved in STAT3-induced radioresistance.
