# Supplementary figures and images for: STAT3 Contributes to Radioresistance in Cancer
Source: Front Oncol. 2020 Jul 7;10:1120. doi: 10.3389/fonc.2020.01120 (PMC7358404; doi:10.3389/fonc.2020.01120)

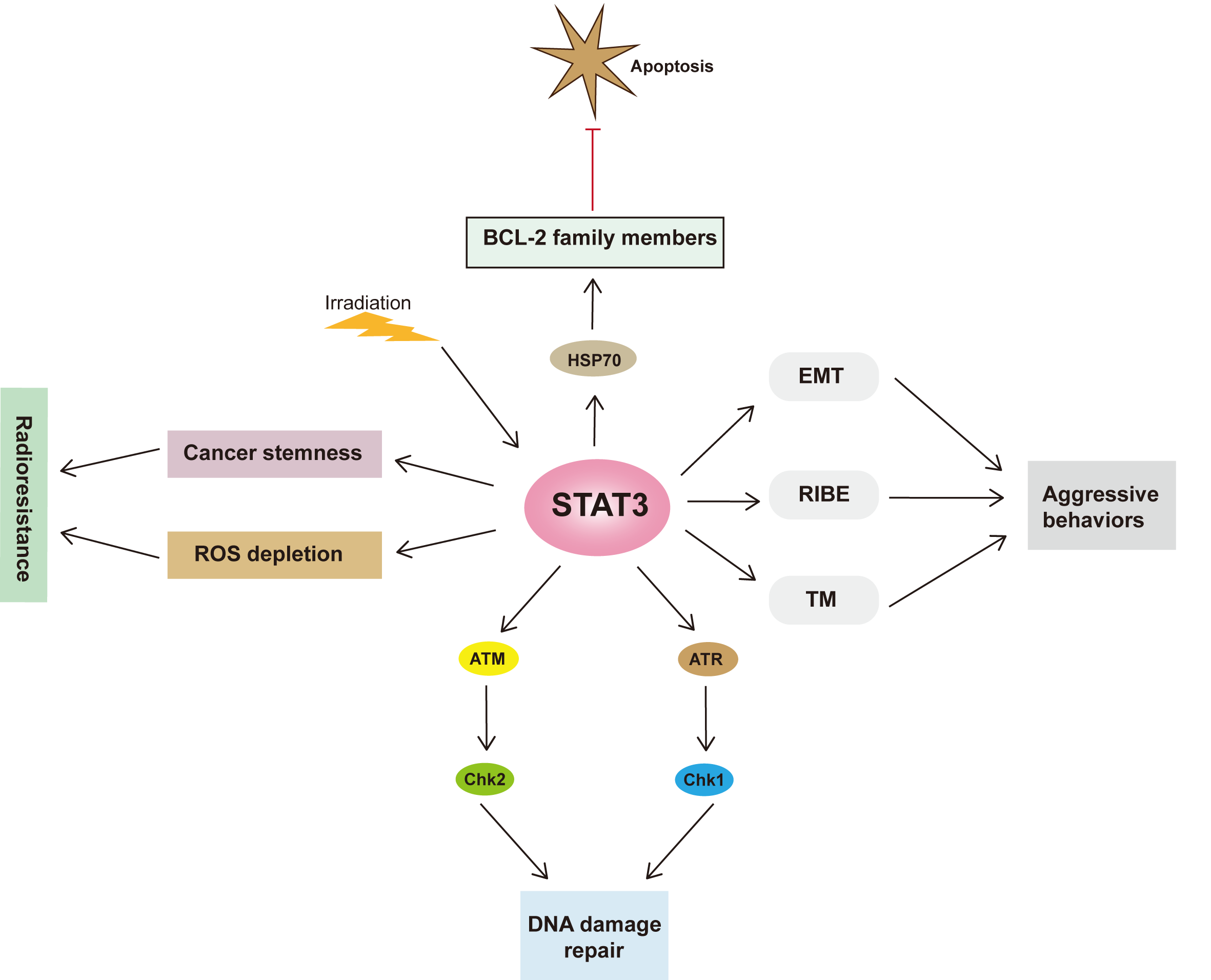

Supplement: Supplementary Figure 1 — Radioresistance caused by signaling pathways related to STAT3. Radiation induced anti-apoptosis is mediated by STAT3-HSP70-BCL2 family members pathways. After radiation treatment, STAT3 promoted aggressive behaviors in tumor cells through epithelial–mesenchymal transition (EMT), radiation-induced bystander effect (RIBE) and tumor microenvironment (TM). STAT3 contributes to DNA damage repair through ATM-Chk2 and ATR-Chk1 pathways. Cancer stemness and reactive oxygen species (ROS) depletion are also involved in STAT3-induced radioresistance. [file Image_1.TIF]
